# Recharacterization data for a geriatric gastrin polycolonal antibody

**DOI:** 10.1016/j.dib.2019.103886

**Published:** 2019-04-01

**Authors:** Steven Dodd, Andrea Varro, D. Mark Pritchard, Graham J. Dockray

**Affiliations:** Department of Cellular and Molecular Physiology, Institute of Translational Medicine, University of Liverpool, Crown St, Liverpool, L69 3BX, UK

**Keywords:** Antibody, Radioimmunoassay validation, Gastrin

## Abstract

Radioimmunoassay data on the recharacterization of a gastrin polycolonal antibody first generated in 1973 is presented. The data include specificity, effect of matrix, and the establishment of a reference range for circulating fasting gastrin concentrations in normal subjects. For a discussion of the interpretation of the data, please see doi, 10.1016/j.peptides.2019.02.001 [1].

**Table:** Specifications table

Subject area	*Physiology*
More specific subject area	*Gut hormone assay*
Type of data	*Radioimmunoassay data in figures and a table*
How data was acquired	*RIASTAR γ-counter (Packard)*
Data format	*Analyzed*
Experimental factors	*The following assay criteria were applied: 1. Non-specific binding (control binding in the absence of antibody) was set to “reject assay” if ratio of “bound” (B) to free (F) label was >0.099. 2. Antibody titration was performed before assays to determine antibody dilution giving B/F of 1.0 in the absence of competing peptide. 3. Batches of*^*125*^*I-labelled heptadecapeptide gastrin were initially evaluated to ensure that maximal binding (antibody bound label in the presence of an excess of antibody) gave a B/F of >4.00.*
Experimental features	*Validation data for a polyclonal antibody to gastrin using*^*125*^*I-labelled heptadecapeptide gastrin in a radioimmunoassay format.*
Data source location	*University of Liverpool, Liverpool, UK.*
Data accessibility	*Data file within this article*
Related research article	G.J. Dockray, Validation of antibody-based assays for regulatory peptides: do it once, get it right, and exploit the under-appreciated benefit of long-term antibody stability Peptides 10.1016/j.peptides.2019.02.001[1]

**Table:** 

**Value of the data** - The data presented here are useful in providing an indication of how antibody validation for radioimmunoassay can be conducted in a manner that endures over many years. - The key features of specificity, sensitivity, reproducibility and effect of matrix can be compared with an original report produced 44 years earlier. - The data should allow researchers to see how they might rigorously characterize their assays in a way that is sustainable for decades [1].

## 1 Data

**Fig. 1:**
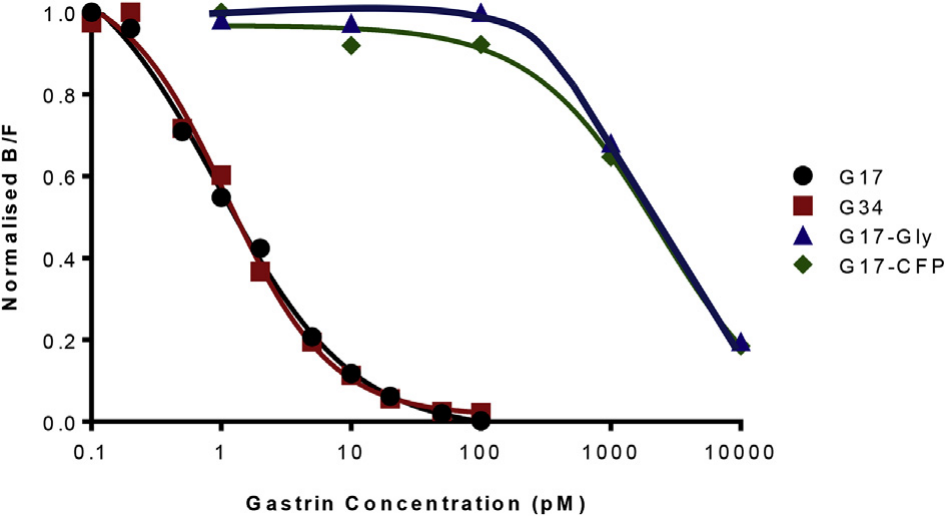
**Antibody L2 is specific for C-terminally amidated gastrins.** Similar curves (expressed as the ratio of antibody bound to free label) showing inhibition of binding of ^125^I-G17 to antibody L2 by human unsulfated heptadecapeptide gastrin (G17) and its NH_2_-terminally extended 34-residue precursor (big gastrin, G34) over the range 0.1–100 pM. Human G17 extended at the COOH-terminus by –Gly (G17-Gly) or –Ser-Ala-Glu-Asp-Glu-Asn (G17-CFP) only inhibited binding at concentrations of 1 nM or higher (ie approximately 1000-fold higher concentrations).

**Fig. 2:**
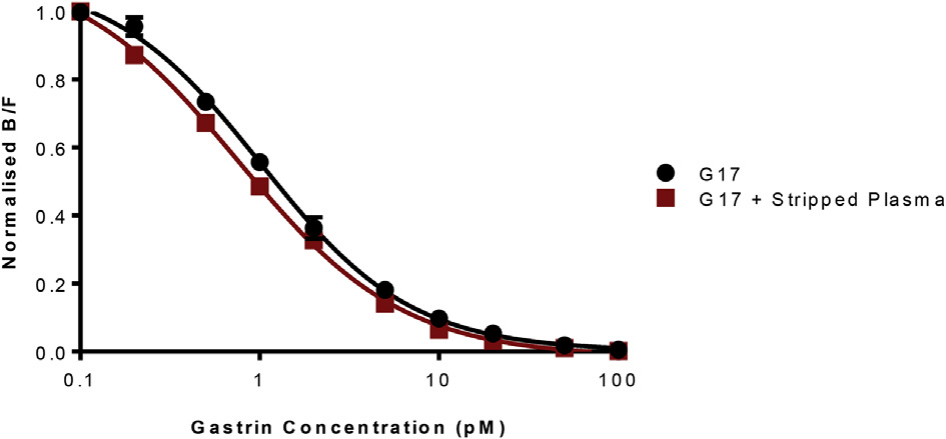
**The effect of hormone-free matrix (human plasma) in radioimmunoassays using antibody L2.** The addition of gastrin-stripped plasma (1:20) had negligible effect on the inhibition of binding of ^125^I-G17 to antibody L2 by human unsulfated heptadecapeptide gastrin (G17) over the range 0.1–100 pM. Mean ± S.E. (n = 3).

**Fig. 3:**
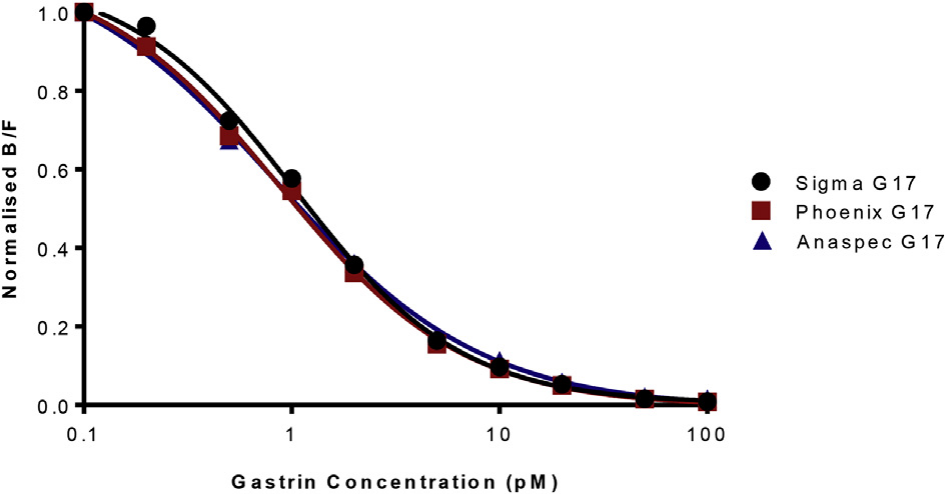
**Three commercially available G17 standards exhibited similar activity with antibody L2.** Synthetic human G17 was sourced from three manufacturers (Sigma, Poole, Dorset; Phoenix, Arizona; Anaspec, Liege, Belgium). The dilution curves were virtually identical.

**Fig. 4:**
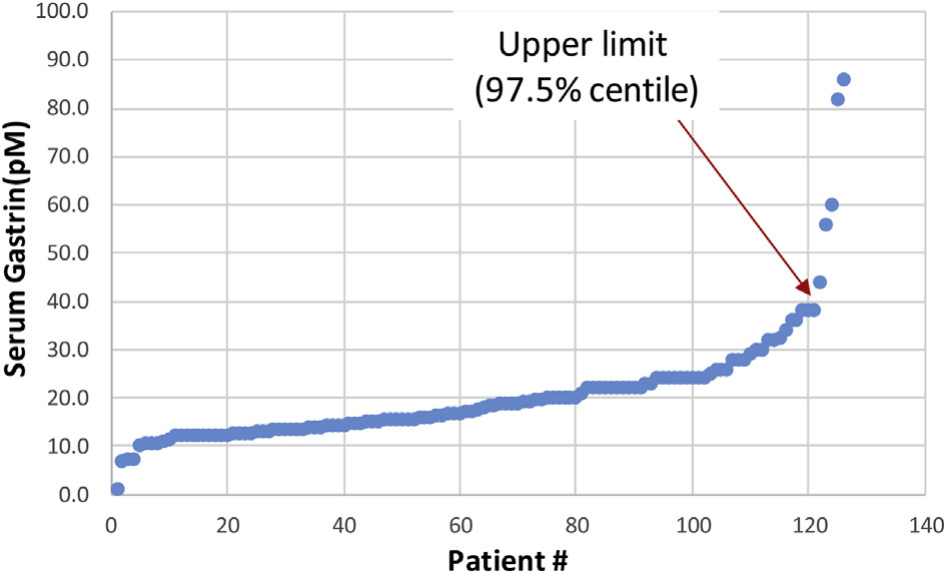
**Reference range for fasting circulating gastrin.** Circulating fasting gastrin concentrations were determined in 126 normal subjects (aged 18–70; 40 male; all *Helicobacter pylori* negative, normal upper gastrointestinal histology, and not taking proton pump inhibitors, H2-receptor antagonists, aspirin or non-steroidal anti-inflammatory drugs). The upper limit of normal is defined as the 97.5 percentile and corresponds to 40pM.

Data are presented that show specificity, effect of matrix, sensitivity, reproducibility and a normal serum concentrations obtained using radioimmunoassay employing a gastrin polyclonal antibody that had been generated 44 years earlier (see accompanying data file). Specificity data (Fig. 1), are presented using a panel of four peptides that in addition to G17 itself, include an N-terminal extended precursor (G34) and two C-terminally modified peptides (G17-CFP, G17-Gly). Data on the effect of matrix (Fig. 2) were obtained by comparison of a standard curve of G17 alone, or together with the addition of hormone-stripped human plasma. Three different synthetic human G17 standards performed similarly (Fig. 3) in the assay. The inter- and intra-assay variations (Table 1) were comparable to those originally described. Data on a reference range for fasting circulating gastrin in normal subjects (Fig. 4) were generated from subjects that were found to be negative for *Helicobacter pylori,* to have normal gastric antral and corpus histology and were not taking drugs known to influence gastric function (proton pump inhibitors, H2-receptor antagonists, aspirin, non-steroidal anti-inflammatory drugs) at the time of sampling.

**Table 1:** **Inter-and intra-assay variation and the limit of detection of gastrin in plasma.** Mean inter- and intra-assay variation for n = 6 replicates expressed as the coefficient of variation. Limit of detection of gastrin in plasma diluted 1:20 in the assay (based on 3x SD for a blank sample).

	Antibody L2
Inter-assay variation	6.3%
Intra-assay variation	15.7%
Limit of detection	2.5 pM

## 2 Experimental design, materials, and methods

### 2.1 Antibody L2

Rabbit polyclonal antibody L2 had been raised in 1973 following immunization with a mixture of porcine unsulfated and sulfated heptadecapeptide gastrin (G17) coupled to bovine serum albumin using carbodiimide as previously described [2], [3]. Aliquots were stored undiluted at −20 °C. A working stock solution was prepared at a dilution of 1:1000 in phosphate buffered saline containing 0.1% bovine serum albumin (Jackson immunoresearch, USA) and 0.1% thimerosal.

### 2.2 Hormone-free matrix

Human-free matrix was generated from 500ml outdated blood bank human plasma (Baxter healthcare, UK) by incubation with 50 g activated charcoal (Sigma-Aldrich) at 4 °C for 30 min and then centrifuged (3000×*g*, 4 °C, 10 min). The supernatant was initially filtered through glass fibre filter paper (Whatman GF/B, Sigma-Aldrich) to remove larger particulates of charcoal then filtered through Sep-Pak C18 cartridges (Waters, UK) that had been primed with 10 mL acetonitrile 50% v/v in distilled water and washed with 10mL 0.02 M sodium barbitone buffer pH 8.2. The plasma was then aliquoted and stored at −20 °C.

### 2.3 Radioimmunoassay

Samples were analysed in duplicate by RIA using a diluent of 0.02 M sodium barbitone, containing 0.5 g/L sodium azide and 0.1% bovine serum albumin (Jackson immunoresearch). The label was [^125^I]Tyr^12^-G17 peptide (Lot. No. CP21770, Perkin Elmer) and antibody was used at a final dilution of 1:350,000. Tubes were incubated for 48 h at 4 °C and free radiolabel was separated from antibody-bound label by addition of 100 μL dextran-coated charcoal. The latter was freshly prepared by adding (dropwise with shaking) to 5 g activated charcoal a solution containing 250 mg dextran (Sigma-Aldrich), followed by another containing 250 mg skimmed milk powder (Marvel International Food Logistics Ltd., UK); the volume was then made up to 50 mL using distilled H_2_O. Samples were centrifuged at 2000×*g* for 10 min at 4 °C. The supernatant was decanted and the radioactivity of both supernatant and pellet counted for 1 min using a Packard Bell RIAstar gamma counter. The ratio of bound to free (B/F) label in the sample minus the non-specific binding of the radiolabel (determined from control tubes in which antibody was omitted) was calculated.

### 2.4 Standard peptides

Standard curves were generated via the serial dilution of synthetic human gastrin peptides. Data are provided for the following synthetic peptides: human non-sulfated G17 (Phoenix Pharmaceuticals, Sigma-Aldrich, and Anaspec), human non-sulfated G34 (Phoenix Pharmaceuticals, USA), G17-CFP (Pepsyn, University of Liverpool, UK) and G17-Gly (Pepsyn, University of Liverpool, UK).

### 2.5 Subjects

Subjects (n = 126) were selected from a cohort of ∼1400 patients, aged 18–70, referred for diagnostic upper gastrointestinal endoscopy and recruited for a study approved by the Liverpool Local Research Ethics Committee and by the Royal Liverpool and Broadgreen University Hospitals NHS Trust; all patients gave written, informed consent. Data described here were obtained from subjects subsequently found to be *H. pylori* negative by antral urease test (Pronto, Medical Instrument, Solothurn, Switzerland), serology and histology; in all selected cases, biopsies of antral and corpus mucosa exhibited normal histology. Exclusion criteria included cancer or upper gastrointestinal preneoplastic disease, diabetes mellitus, coma or hemodynamic instability, being moribund or having terminal malignancy, cirrhosis (Child B or C), abnormal clotting or bleeding diathesis, inability to give informed consent, contraindication to endoscopy, pregnancy, HIV, hepatitis B or C infections. No selected patients were taking proton pump inhibitors, H-2 receptor antagonists, aspirin or non-steroidal anti-inflammatory drugs. Venous blood samples were drawn into 7.5 mL Sarstedt S-Monovette^®^ tubes with clotting activator (Sarstedt, Germany) and stored on ice immediately until centrifuged (2500×*g*, 10 min, 4 °C) and the supernatant separated and stored at −20 °C.
